# Efficacy and safety of Xiangsha liujunzi decoction for functional dyspepsia: a systematic review and meta-analysis

**DOI:** 10.3389/fphar.2024.1356899

**Published:** 2024-06-12

**Authors:** Longhua Wang, Xia Ding, Xinning Yao, Ping Li, Fuwen Zhang, Fenglei Wang, Bai Li, Jing Li

**Affiliations:** ^1^ Department of Gastroenterology, Dongzhimen Hospital, Beijing University of Chinese Medicine, Beijing, China; ^2^ School of Traditional Chinese Medicine, Beijing University of Chinese Medicine, Beijing, China

**Keywords:** effectiveness, functional dyspepsia, Xiangsha liujunzi decoction, systematic review, meta-analysis

## Abstract

**Background:**

Functional dyspepsia is a highly prevalent digestive disorder. The limited effectiveness of current pharmaceutical interventions necessitates the exploration of alternative therapeutic options for functional dyspepsia. Xiangsha liujunzi decoction, a well-known traditional Chinese medicine formulation, has been widely employed in the treatment of functional dyspepsia in China. Nevertheless, the effectiveness of Xiangsha liujunzi decoction in the treatment of functional dyspepsia remains uncertain.

**Objective:**

To examine the effectiveness and safety of Xiangsha liujunzi decoction for treating functional dyspepsia.

**Methods:**

We retrieved seven databases containing randomized controlled trials on functional dyspepsia published up until 31 July 2023. The quality of these studies was evaluated using the Cochrane Risk of Bias assessment tool. The analysis of data was performed using the software RevMan 5.4. The total clinical effectiveness rate was evaluated as the primary outcome. In addition, gastric emptying rate, symptom score and safety evaluation were evaluated as the secondary outcomes.

**Results:**

The meta-analysis included 23 studies, involving 2,101 individuals. Xiangsha liujunzi decoction demonstrated a significantly higher clinical effectiveness rate compared to the control group (RR 1.27; 95% CI 1.21, 1.33; *p* < 0.00001). Moreover, it exhibited superior gastric emptying rate and symptom score improvement compared to the control group. Nevertheless, no remarkable differences were detected in safety between Xiangsha liujunzi decoction and the control group (RR 0.67; 95% CI 0.16, 2.76; *p* = 0.58).

**Conclusion:**

The findings of this study suggest that Xiangsha liujunzi decoction exhibits effectiveness and no significant adverse events observed. However, because of the low quality of the enrolled studies, more high-quality and strict design randomized controlled trials are required in the future.

## 1 Introduction

Functional dyspepsia (FD) is a prevalent functional gastrointestinal disorder (Drossman et al., 1993; von Wulffen et al., 2019), characterized by one or more upper gastrointestinal symptoms such as postprandial fullness, early satiety, epigastric pain and epigastric burning without any identifiable organic causes according to the Rome IV (Stanghellini et al., 2016). It can be categorized into two distinct subtypes: postprandial distress syndrome (PDS) and epigastric pain syndrome (EPS) (Stanghellini et al., 2016). The worldwide occurrence of FD varies between 7% and 15% (Zand Irani et al., 2021; Jones et al., 2022; Kovács et al., 2022), with approximately 10% of adults meeting the diagnostic criteria for FD based on Rome Ⅳ guidelines (Aziz et al., 2018). The underlying mechanisms of FD remain poorly comprehended and encompass various factors such as compromised gastric accommodation, delayed emptying of the stomach, heightened sensitivity in the digestive system, secretion of gastric acid, genetic predisposition, early-life experiences, lifestyle factors, minor inflammation in the duodenum, and infection caused by *Helicobacter pylori* (*H. pylori*) (Black et al., 2022; Miwa et al., 2022). Despite the utilization of various therapies for FD, such as *H. pylori* eradication therapy, antisecretory therapy, prokinetic agents and psychotropic agents, the treatment of FD often remains unsatisfactory due to limited understanding of its underlying mechanisms (Camilleri and Stanghellini, 2013). Furthermore, the persistent symptoms of FD not only have negative impacts on psychological well-being and quality of life but also impose a significant financial burden on individuals (Aro et al., 2011). Therefore, it is crucial to explore new complementary and alternative therapies like botanical drugs in order to enhance the clinical effectiveness of FD.

Xiangsha liujunzi decoction (XSLJZD), a renowned traditional Chinese medicine (TCM) formula extensively employed in China for the management of FD (Zhang and Zhao, 2017), comprises eight botanical drugs: *Panax ginseng* C.A.Mey. [Araliaceae], *Atractylodes macrocephala* Koidz. [Asteraceae], *Poria cocos* (Schw.) Wolf [Polyporaceae], *Dolomiaea costus* (Falc.) Kasana and A.K.Pandey [Asteraceae], *Wurfbainia villosa* (Lour.) Škorničk. and A.D.Poulsen [Zingiberaceae], *Pinellia ternata* (Thunb.) Makino [Araceae], *Citrus reticulata* Blanco [Rutaceae], and *Glycyrrhiza glabra* L. [Fabaceae] (Yue et al., 2021). Based on TCM theory, XSLJZD can enhance spleen function, boost Qi levels, alleviate dampness, and regulate gastric activity. Previous studies have demonstrated that XSLJD could improve gastrointestinal motility disorder in patients with FD exhibiting spleen deficiency syndrome (Zhang J. et al., 2022), as well as effectively inhibit low-grade duodenal inflammation (Zhao et al., 2023a; Zhao et al., 2023b).

However, the effectiveness of Xiangsha liujunzi decoction for treating FD remains unkonwn. Henceforth, it holds great clinical significance to determine both the clinical effectiveness and safety profile of XSLJZD. The objective of this study was thus aimed at determining both the clinical effectiveness and safety profile associated with using XSLJZD for treating patients with FD so as to establish compelling therapeutic evidence supporting its use.

## 2 Materials and methods

XSLJZD contains different botancial drugs in varying proportions: the dried root of *Panax ginseng* C.A.Mey. [Araliaceae; Ginseng radix et rhizoma], the dride rhizome of *Atractylodes macrocephala* Koidz. [Asteraceae; Atractylodis macrocephalae rhizoma], the dried sclerotium of *Poria cocos* (Schw.) Wolf [Polyporaceae; Poria cocos], the dried root of *Dolomiaea costus* (Falc.) Kasana and A.K.Pandey [Asteraceae; Aucklandiae radix], the dried ripe fruit of *Wurfbainia villosa* (Lour.) Škorničk. and A.D.Poulsen [Zingiberaceae; Amomi fructus], the dried tuber of *Pinellia ternata* (Thunb.) Makino [Araceae; Pinelliae rhizoma], the dried pericarp of the ripe fruit of *Citrus reticulata* Blanco [Rutaceae; Citri reticulatae pericarpium], and the dried root and rhizome of *Glycyrrhiza glabra* L. [Fabaceae; Glycyrrhizae radix et rhizoma praeparata cum melle] (Table 1).

**TABLE 1:** Composition of botanical drugs in XSLJZD.

Scientific name	Family	Species name	Part(s) of drug used
*Panax ginseng* C.A.Mey	Araliaceae	Ginseng radix et rhizoma	Dried root
*Atractylodes macrocephala* Koidz	Asteraceae	Atractylodis macrocephalae rhizoma	Dride rhizome
*Poria cocos* (Schw.) Wolf	Polyporaceae	Poria cocos	Dried sclerotium
*Dolomiaea costus* (Falc.) Kasana and A.K.Pandey	Asteraceae	Aucklandiae radix	Dried root
*Wurfbainia villosa* (Lour.) Škorničk. and A.D.Poulsen	Zingiberaceae	Amomi fructus	Dried ripe fruit
*Pinellia ternata* (Thunb.) Makino	Araceae	Pinelliae rhizoma	Dried tuber
*Citrus reticulata* Blanco	Rutaceae	Citri reticulatae pericarpium	Dried pericarp of the ripe fruit
*Glycyrrhiza glabra* L.	Fabaceae	Glycyrrhizae radix et rhizoma praeparata cum melle	Dried root and rhizome

This study was registered with PROSPERO (No. CRD42023446320; http://www.crd.york.ac.uk/prospero) and conducted in accordance with the guidelines known as PRISMA, which provide recommendations for reporting systematic reviews and meta-analyses (Page et al., 2021).

### 2.1 Inclusion and exclusion criteria

#### 2.1.1 Patients

Adult patients who meet the Rome criteria for FD were included in this study without restrictions on sex or region.

#### 2.1.2 Interventions

Studies on XSLJZD or modified XSLJZD were eligible, while those combined with other interventions such as acupuncture or Western medicine were excluded. The minimum treatment duration was 4 weeks.

#### 2.1.3 Control groups

The control group was administered with Western medicine, Chinese patent drugs, or placebo.

#### 2.1.4 Outcome measures

Our primary outcome measure was the total clinical effectiveness rate, which is an efficacy index based on the change in symptom scores before and after treatment, encompassing clinical recovery, significant improvement, effectiveness, and ineffectiveness as per the guiding principles of clinical research on new Chinese medicine. The secondary outcomes included safety evaluation, improvement of gastric emptying rate and symptom score improvement.

#### 2.1.5 Study designs

Randomized controlled trials (RCTs) published in Chinese or English before 31 July 2023 were considered eligible.

### 2.2 Search strategy

A comprehensive search across multiple databases was performed up to 31 July 2023, including EMBASE, Cochrane Library, PubMed, China National Knowledge Infrastructure (CNKI), Chinese Scientific Journals Database (VIP), Wanfang database, and Chinese Biological Medical Database (CBM).

Two reviewers (Longhua Wang and Xinning Yao) independently screened all citations using Mesh terms and free terms retrieval methods. The searching strategies for PubMed were comprehensively outlined in Table 2.

**TABLE 2:** PubMed search methodology and strategy.

No.	Search items
#1	Dyspepsia [MeSH Terms]
#2	Dyspepsia [Title/Abstract] OR dyspeptic [Title/Abstract] OR NUD [Title/Abstract] OR FD [Title/Abstract] OR EPS [Title/Abstract] OR PDS [Title/Abstract]
#3	Indigestion [Title/Abstract] OR indigestive [Title/Abstract]
#4	#1 OR #2 OR #3
#5	xiang-sha-liu-jun-zi decoction [Title/Abstract] OR xiang sha liu jun zi decoction [Title/Abstract] OR xiang sha liu jun zi tang [Title/Abstract] OR xiangshaliujunzi [Title/Abstract] OR XSLJZ [Title/Abstract] OR xiangsha liujunzi [Title/Abstract]
#6	#4 AND #5

### 2.3 Study selection and data extraction

Two researchers, Longhua Wang and Xinning Yao, individually utilized Endnote X9.1 to select the studies. They carefully examined the title, abstract, and full text of each study based on predefined criteria for inclusion and exclusion. In case of any disagreement between the two researchers, a third researcher named Xia Ding acted as an arbitrator.

Subsequently, Longhua Wang and Ping Li independently extracted relevant information from selected studies including literature title, first author, sample size, publication year, diagnostic criteria, interventions, duration of treatment, indicators for evaluating outcomes, findings, duration of follow-up and adverse events. Any discrepancies were resolved by a final decision made by Fenglei Wang.

### 2.4 Quality assessment

The risk of bias was independently assessed by two researchers (Longhua Wang and Ping Li) using the Cochrane risk of bias tool. The evaluation encompassed seven aspects, namely the generation of random sequences, concealment of allocation, blinding of participants and personnel, blinding of outcome assessment, incomplete outcome data, selective reporting, and other biases. A low, high, or unclear risk assessment was assigned for each item. In case of the researchers, a final decision was reached through discussion.

### 2.5 Data analysis and synthesis

The analysis of data was conducted utilizing RevMan 5.4.1 software. For dichotomous data, relative risks (RR) with corresponding 95% confidence intervals (CI) were used; for continuous data standardized mean difference (SMD) with corresponding 95% CI were examined. Heterogeneity was assessed using *χ* ^2^ test and inconsistency index statistic (*I* ^2^). If substantial heterogeneity was present (*I* ^2^ > 50% or *p* < 0.05), a random effects model was applied to compute the pooled RR; Conversely, in the absence of significant heterogeneity, a fixed effects model was utilized instead. Sensitivity analysis examined potential sources of heterogeneity and assessed result robustness. Statistically significance for pooled results was when *p* < 0.05. In addition, funnel plots were utilized to assess publication bias.

## 3 Results

### 3.1 Study selection

**FIGURE 1:**
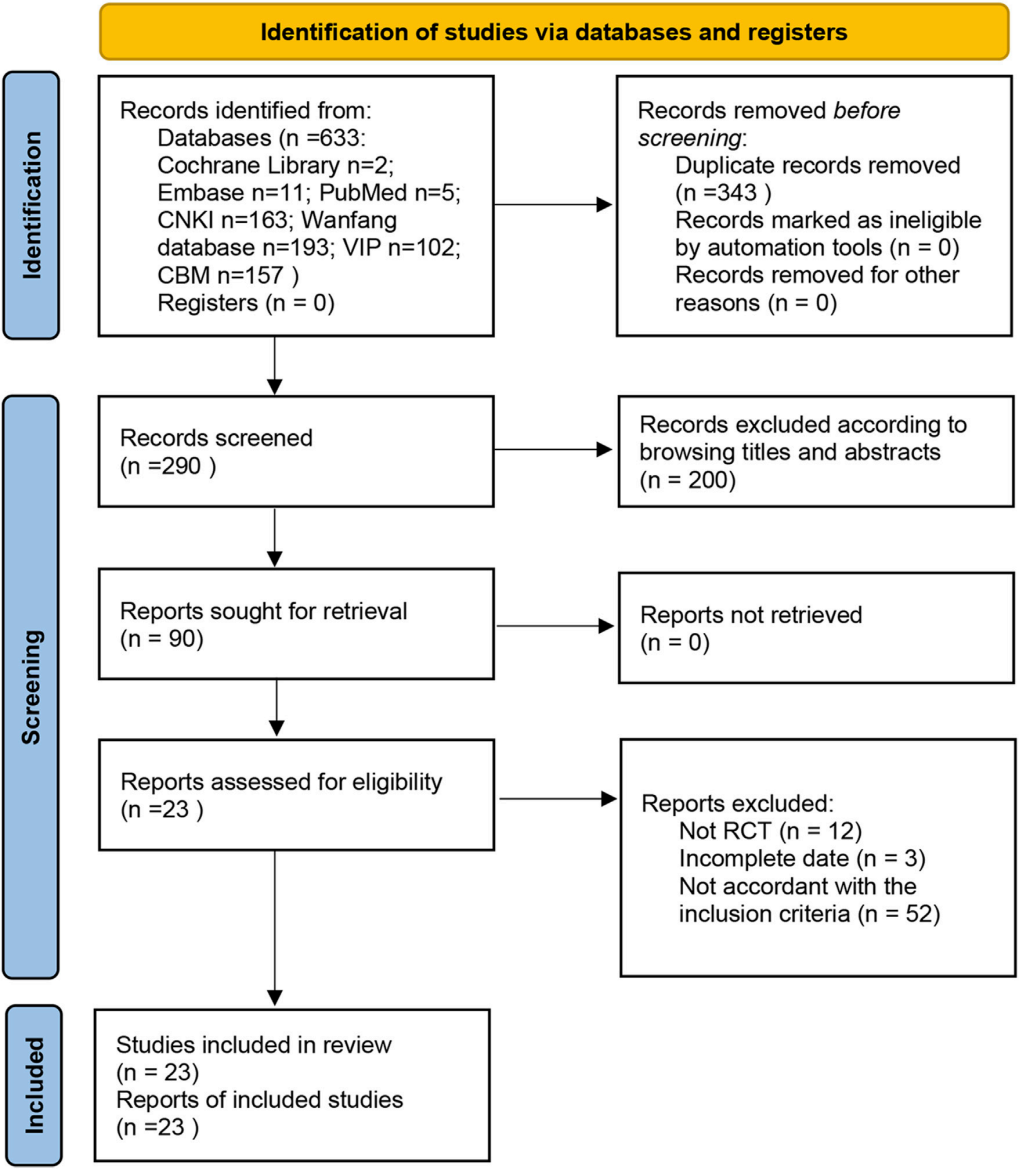
Flow chart of study screening.

The search strategy identified a total of 633 studies from databases, including 5 in PubMed, 11 in Embase, 2 in the Cochrane Library, 163 in CNKI, 193 in Wanfang, 102 in VIP, and 157 in CBM. Among these studies, there were duplicates (*n* = 343), irrelevant studies excluded based on title and abstract screening (*n* = 200), non-RCTs (*n* = 12), incomplete date (*n* = 3), and studies not meeting inclusion criteria (*n* = 52). Ultimately, a total of 23 RCTs conducted exclusively in China met the inclusion criteria for this meta-analysis (Figure 1).

### 3.2 Description of studies

Of 2,101 patients with FD: experiment groups included 1,081 cases while control groups had 1,020 cases. XSLJZD or modified XSLJZD was administered solely to the experiment groups whereas Western medicine, placebo, or Chinese patent drugs were used as controls. Detailed characteristics of the included studies can be found in Supplementary Table S1.

### 3.3 Risk of bias assessment

**FIGURE 2:**
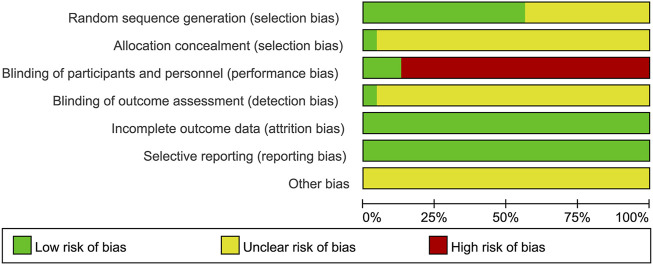
Risk of bias graph.

**FIGURE 3:**
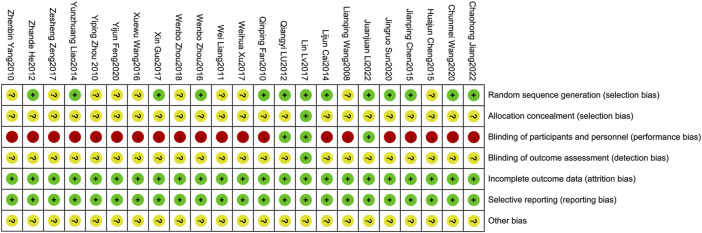
Risk of bias summary.

Methodological quality displayed in Table 3. Figures 2, 3 describe assessment of risk bias.

**TABLE 3:** Assessment of the methodological quality.

First author, year	Randomization	Allocation concealment	Blinding	Cases dropped out	Follow up	Adverse reactions
Zhou (2010)	Random	N.R	N.R	No	N.R	N.R
Zhou et al. (2016)	Random number table	N.R	N.R	No	N.R	No
Yang et al. (2010)	Random	N.R	N.R	No	N.R	4 cases in C
Xu et al. (2017)	Random	N.R	N.R	1 case in E and 2 cases in C	N.R	2 cases in E and 1 case in C
Wang and Yang (2016)	Random	N.R	N.R	No	1 mo	N.R
Wang et al. (2008)	Random	N.R	N.R	No	N.R	N.R
Wang (2020)	Random number table	N.R	N.R	No	N.R	N.R
Sun (2017)	Random number table	N.R	N.R	No	N.R	N.R
Liao (2014)	Random number table	N.R	N.R	No	N.R	N.R
Lu and Hong (2012)	Random number table	N.R	Single-blind	No	N.R	N.R
Liang (2011)	Random	N.R	N.R	No	N.R	N.R
Li et al. (2022)	Computer-generated randomization	N.R	Double-blind	4 cases in E and 2 cases in C	N.R	1 case in E
Jiang (2022)	Random number table	N.R	N.R	No	N.R	N.R
Cai et al. (2014)	Random number table	N.R	N.R	4 cases in E and 3 cases in C	N.R	1 case in E
He (2012)	Random number table	N.R	N.R	No	1 mo	N.R
Guo and Chen (2017)	Random number table	N.R	N.R	No	N.R	N.R
Feng (2020)	Random	N.R	N.R	No	N.R	2 cases in E and 10 cases in C
Fan (2010)	Random number table	N.R	N.R	2 cases in E and 2 cases in C	N.R	No
Cheng and Zhu (2015)	Random	N.R	N.R	No	N.R	N.R
Chen (2015)	Random draw	N.R	N.R	No	N.R	No
Zeng and Tang (2017)	Random	N.R	N.R	No	N.R	N.R
Zhou et al. (2018)	Random	N.R	N.R	No	1 mo	N.R
Lv et al. (2017)	Computer-generated randomization	Opaque envelope	Double-blind	9 cases in E and 5 cases in C	1 mo	No

Annotation: N.R, not reported, E, experiment group, C, control group.

#### 3.3.1 Random sequence generation

Ten studies (Fan, 2010; He, 2012; Lu and Hong, 2012; Cai et al., 2014; Liao, 2014; Zhou et al., 2016; Guo and Chen, 2017; Sun, 2017; Wang, 2020; Jiang, 2022) employed random number tables which were evaluated as having low risk of bias. Two studies (Lv et al., 2017; Li et al., 2022) utilized computer-generated randomization were also considered to have low risk of bias. However, the remaining studies were deemed to have an unclear risk of bias due to lack of sufficient description regarding the randomization method.

#### 3.3.2 Allocation concealment

The study of Lin Lv et al. (2017) provided a description on how the allocation concealment was done, resulting in a low risk of bias. However, other studies did not elaborate on this aspect, hence an unclear risk of bias was assigned.

#### 3.3.3 Blinding of participants and personnel

Two studies employed a “double-blind” methodology (Lv et al., 2017; Li et al., 2022), with Lin Lv’s study (Lv et al., 2017) providing detailed information on the blinding process, resulting in a low risk of bias assessment. Nevertheless, it should be emphasized that the remaining twenty-one studies did not provide specific descriptions regarding blinding methods. Due to significant differences between XSLJZD and control groups, achieving blinding among patients and personnel was challenging, leading to a high risk of bias assessment.

#### 3.3.4 Blinding of outcome assessment

The study conducted by Lv et al. (2017) was deemed to have a low risk of bias, while the remaining studies lacked descriptions regarding blinding of outcome assessments, resulting in an unclear risk of bias being assessed.

#### 3.3.5 Incomplete outcome data

The absence of any missing or incomplete outcomes in all included studies resulted in a low risk of bias.

#### 3.3.6 Selective reporting

The studies were considered to have a low risk of bias as they accurately presented the outcomes outlined in the Methods section.

#### 3.3.7 Other biases

The presence of research design limitations and insufficient information in each study posed challenges in accurately accessing the risk of bias, thereby resulting in an ambiguous evaluation across all studies.

### 3.4 Primary outcome: the total clinical effectiveness rate

**FIGURE 4:**
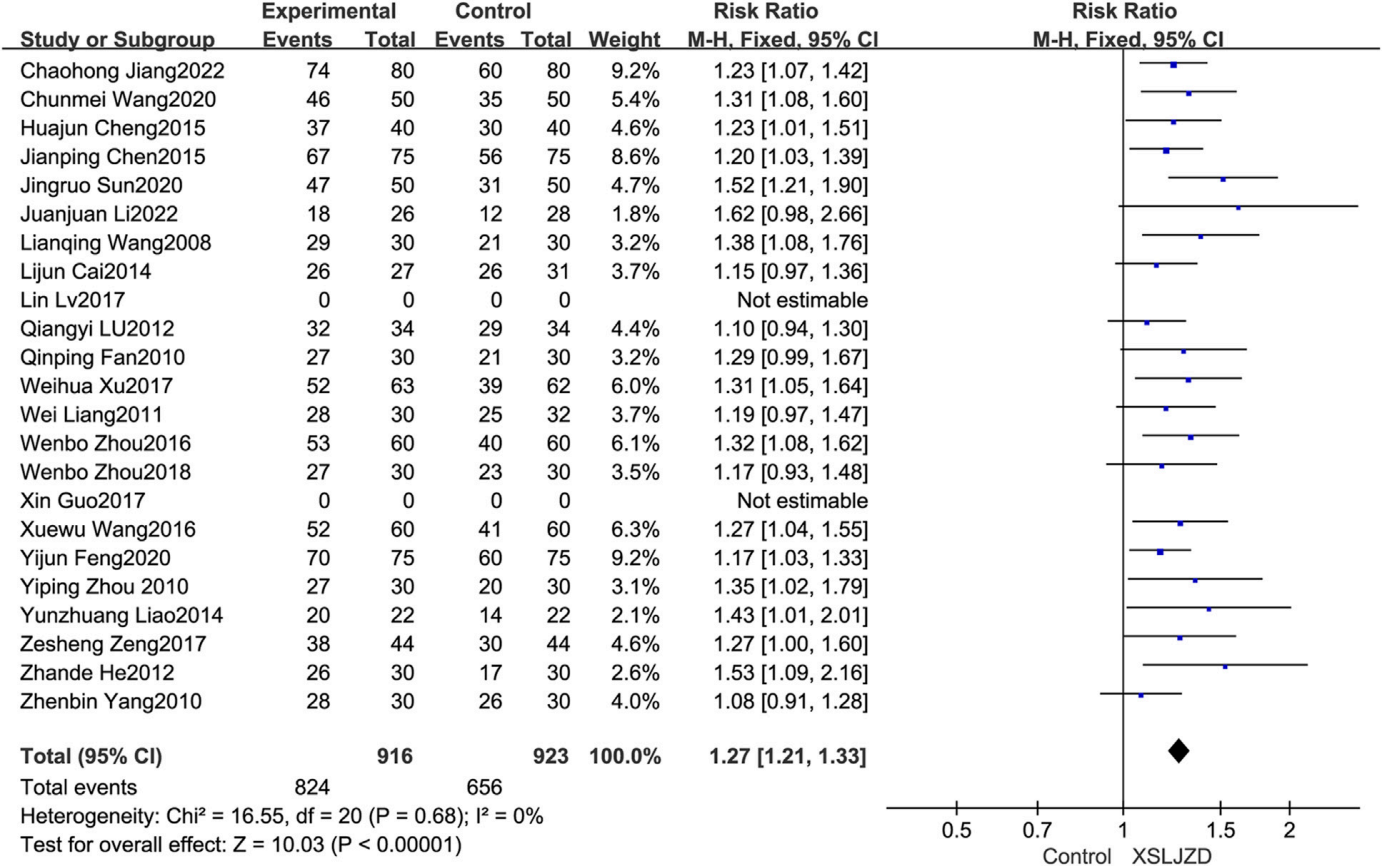
Forest plot illustrating the clinical efficacy.

The total clinical effectiveness rate was described in twenty-one studies (Wang et al., 2008; Fan, 2010; Yang et al., 2010; Zhou, 2010; Liang, 2011; He, 2012; Lu and Hong, 2012; Cai et al., 2014; Liao, 2014; Chen, 2015; Cheng and Zhu, 2015; Wang and Yang, 2016; Zhou et al., 2016; Sun, 2017; Xu et al., 2017; Zeng and Tang, 2017; Zhou et al., 2018; Feng, 2020; Wang, 2020; Jiang, 2022; Li et al., 2022). XSLJZD demonstrated a significantly higher clinical effectiveness rate compared to the control group (RR 1.27; 95% CI 1.21, 1.33; *p* < 0.00001), with low heterogeneity observed (*p* = 0.68, *I* ^2^ = 0%) (Figure 4).

#### 3.4.1 Subgroup analysis

**FIGURE 5:**
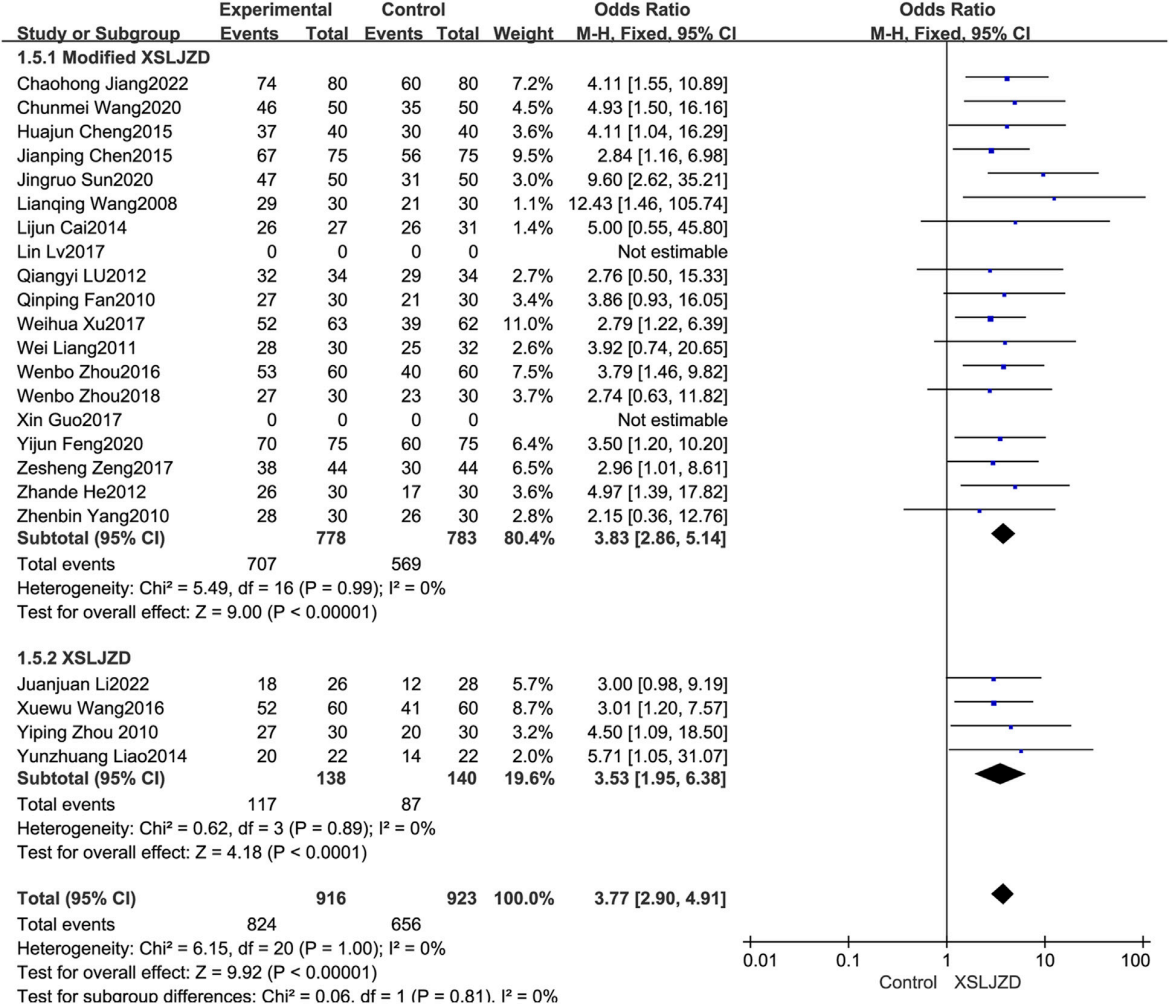
Forest plot of subgroup analysis illustrating the clinical efficacy.

The XSLJZD and modified XSLJZD were both integrated into the experiment group in this review. The study thus conducted an additional subgroup analysis to investigate the disparities and heterogeneity. Modified XSLJZD also demonstrated a significantly higher clinical effectiveness rate compared to the control group (RR 3.83; 95% CI 2.86, 5.14; *p* < 0.00001), with low heterogeneity observed (*p* = 0.99, *I* ^2^ = 0%) (Figure 5).

### 3.5 Publication bias

**FIGURE 6:**
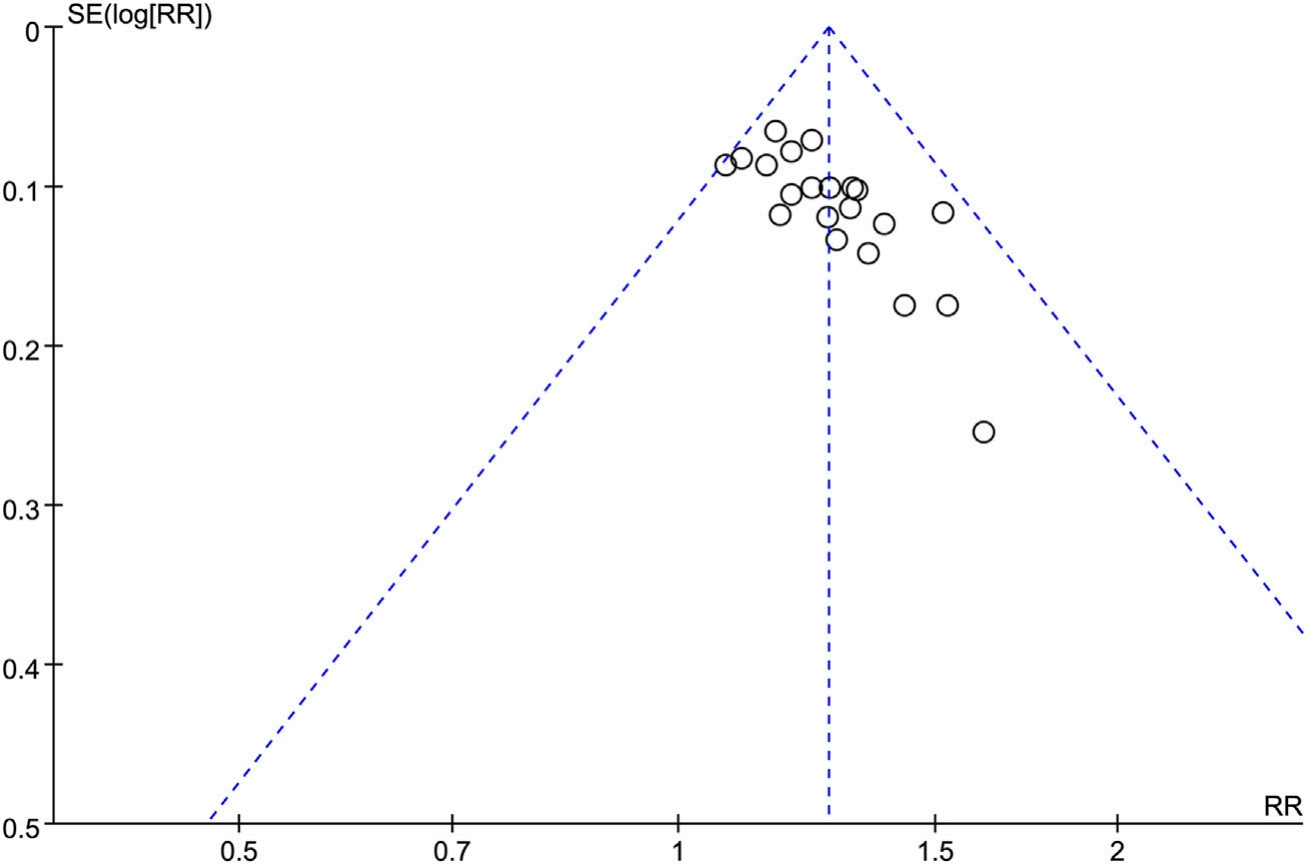
Funnel plot illustrating the clinical efficacy outcomes.

The potential publication bias of the twenty-one studies (Wang et al., 2008; Fan, 2010; Yang et al., 2010; Zhou, 2010; Liang, 2011; He, 2012; Lu and Hong, 2012; Cai et al., 2014; Liao, 2014; Chen, 2015; Cheng and Zhu, 2015; Wang and Yang, 2016; Zhou et al., 2016; Sun, 2017; Xu et al., 2017; Zeng and Tang, 2017; Zhou et al., 2018; Feng, 2020; Wang, 2020; Jiang, 2022; Li et al., 2022) were evaluated using a funnel plot analysis (Figure 6). According to the results obtained from the analysis of the funnel plot in this research, no significant evidence of potential publication bias was observed.

### 3.6 Secondary outcomes

#### 3.6.1 Improvement of gastric emptying rate

**FIGURE 7:**
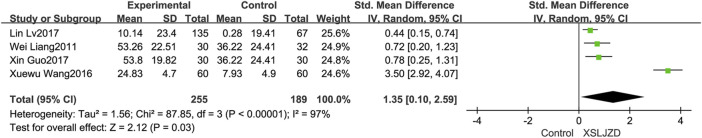
Forest plot illustrating the enhancement of gastric emptying rate.

Four studies (Liang, 2011; Wang and Yang, 2016; Guo and Chen, 2017; Lv et al., 2017) have reported a significant improvement in gastric emptying rate among patients with FD following treatment. XSLJZD demonstrated a significantly higher gastric emptying rate compared to the control group (RR 1.35; 95% CI 0.10, 2.59; *p* = 0.03), although there was substantial heterogeneity observed (*I* ^2^ = 97%, *p* < 0.00001) (Figure 7).

#### 3.6.2 Sensitivity analysis

**FIGURE 8:**
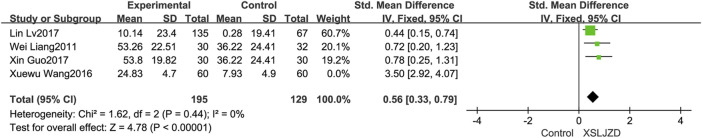
Forest plot illustrating the improvement in gastric emptying rate after excluding a specific study.

The robustness of the outcomes and sources of the heterogeneity were assessed through a sensitivity analysis by systematically eliminating each study. The heterogeneity showed significantly low (*I* ^2^ = 0%, *p* = 0.44) after excluding the study (Wang and Yang, 2016). Despite this, XSLJZD still exhibited a higher gastric emptying rate compared to the control group (RR 0.56; 95% CI 0.33, 0.79; *p* < 0.00001) (Figure 8). Therefore, it can be concluded that the study (Wang and Yang, 2016) contributed as an underlying source of heterogeneity.

#### 3.6.3 Improvement of symptom score

**FIGURE 9:**
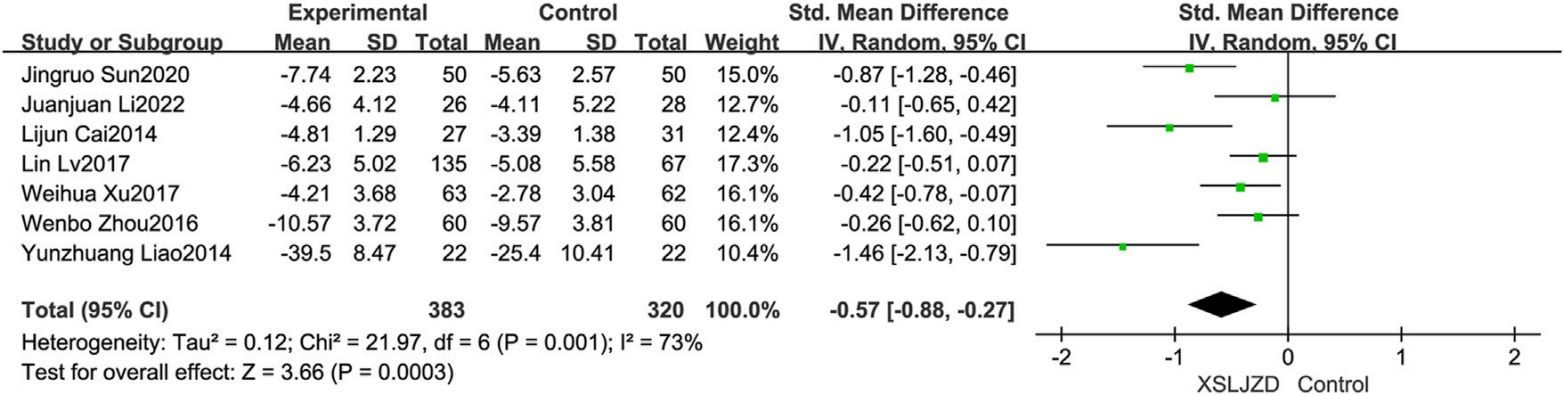
Forest plot illustrating the improvement in symptom scores.

Seven studies (Cai et al., 2014; Liao, 2014; Zhou et al., 2016; Lv et al., 2017; Sun, 2017; Xu et al., 2017; Li et al., 2022) reported the symptom score. XSLJZD exhibited a significantly greater improvement in symptom scores compare to the control group (RR -0.57; 95% CI −0.88, −0.27; *p* = 0.0003), although there was substantial heterogeneity observed (*I* ^2^ = 73%, *p* = 0.001) (Figure 9).

#### 3.6.4 Safety evaluation

**FIGURE 10:**
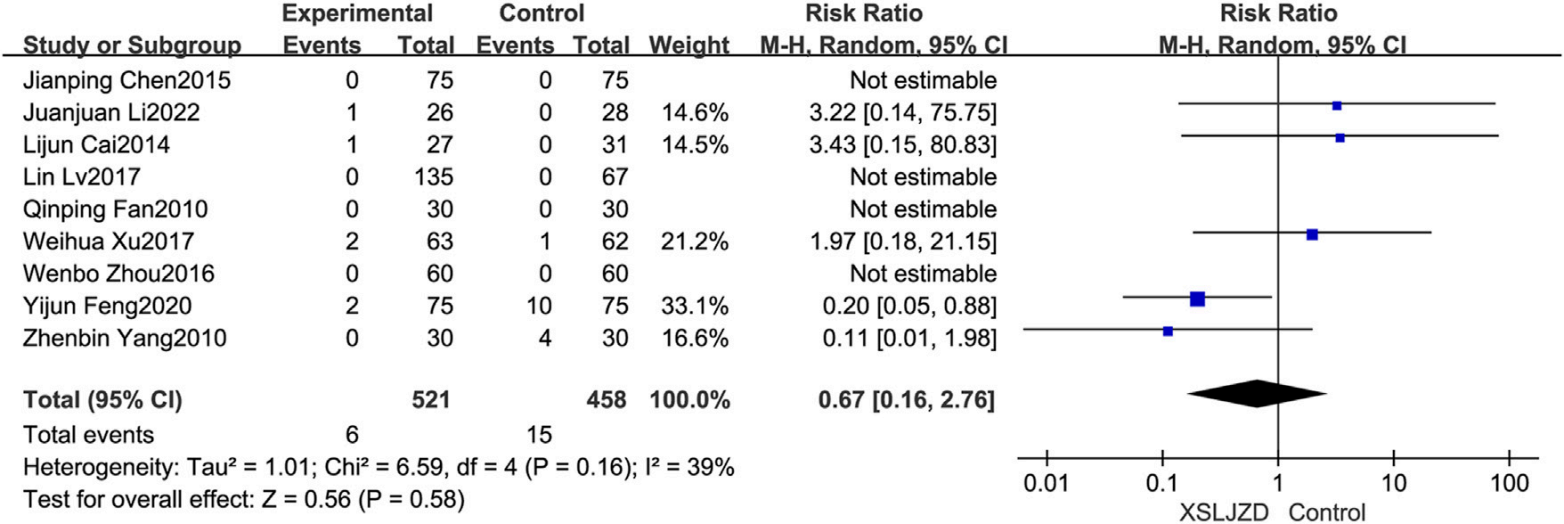
Forest plot depicting the safety assessment findings.

Nine studies reported adverse events (Fan, 2010; Yang et al., 2010; Cai et al., 2014; Chen, 2015; Zhou et al., 2016; Lv et al., 2017; Xu et al., 2017; Feng, 2020; Li et al., 2022). However, no adverse events were described in the remaining studies. Importantly, none of the studies identified any severe adverse events. XSLJZD exhibited comparable safety assessment to the control group with no significant differences observed (RR 0.67; 95% CI 0.16, 2.76; *p* = 0.58) and low heterogeneity was observed (*I* ^2^ = 39%, *p* = 0.16) (Figure 10).

## 4 Discussion

### 4.1 Summary of evidence

FD is a highly prevalent digestive disorder with an intricate pathophysiology that hampers effective treatment strategies (Ford et al., 2020). Consequently, there is a pressing need to explore novel complementary and alternative therapies for FD. Furthermore, no comprehensive systematic review or meta-analysis on the efficacy of XSLJZD for FD has been published in English literature. Notably, XSLJZD has gained widespread usage in China as a therapeutic approach for FD. In this review, we performed a meta-analysis aiming to evaluate the efficacy of XSLJZD in managing FD symptoms. Our findings revealed that: 1) XSLJZD exhibited significantly superior clinical efficacy compared to the control group; 2) XSLJZD demonstrated notable improvements in gastric emptying rate and symptom scores.

According to the pharmacology of herbal medicine, *Dolomiaea costus* (Falc.) Kasana and A.K.Pandey has been reported to enhance gastrointestinal motility, protect gastric mucosa, and exhibit anti-ulcer and anti-inflammatory effects (Zheng et al., 2022). *Wurfbainia villosa* (Lour.) Škorničk. and A.D.Poulsen has demonstrated significant potential in promoting gastric emptying, enhancing gastrointestinal peristalsis for gastroprotection, and exerting anti-ulcer properties (Lu et al., 2016). *Panax ginseng* C.A.Mey is known for its ability to invigorate vital energy and benefit the spleen and lung. Total ginsenosides have shown promising results in improving gastrointestinal motility in rats with FD by modulating gastrointestinal hormone levels and alleviating motility disorders (Lin et al., 2020). *Poria cocos* (Schw.) Wolf can enhance the reparative capacity of gastrointestinal mucosa by modulating immune and gastrointestinal functions (Zuo et al., 2023). *Atractylodes macrocephala* Koidz exerts gastroprotective effects through bidirectional regulation of gastrointestinal motility and modulation of intestinal microecology (Zhang W. X. et al., 2022). *Pinellia ternata* (Thunb.) Makino significantly promotes gastrointestinal motility and regulates gastric acid secretion to safeguard gastric mucosa integrity (Li et al., 2021). *Citrus reticulata* Blanco can effectively improve digestion and alleviate symptoms of indigestion (Xu et al., 2022).

Numerous researchers have reported the effectiveness of XSLJZD for treating FD, which aligns with our findings. XSLJZD demonstrated a significant improvement in gastrointestinal motility with the restoration of mitochondrial quality control system by inhibiting PINK1/Parkin-mediated mitophagy and division (Zhang J. et al., 2022). Additionally, XSLJZD was found to downregulate TRPV1 and 5-HT expression levels in the duodenum of rats with FD, thereby alleviating gastric hypersensitivity, which may contribute to its therapeutic mechanisms for FD (Zhao et al., 2019). Through network pharmacology analysis and experimental validation (Zhao et al., 2023b), it was observed that XSLJZD effectively reduced p-ERK, p-P38MARK, as well as downstream p-NF-κBp65 levels to modulate low-grade duodenal inflammation and ameliorate FD symptoms. Importantly, recent understanding of FD pathophysiology suggests that low-grade duodenal inflammation plays a central role (Miwa et al., 2019).

### 4.2 Limitations of our reviews

This review has several limitations. First of all, the included studies exhibited low quality, potentially introducing a high-risk bias across all enrolled studies. Secondly, most studies lacked published protocols or sufficient information to assess the risk of bias adequately. The inclusion of all studies did not adhere to double-blinding protocols, specifically due to the inherent differences in external characteristics between herbal decoctions and Western medicine. Therefore, it is imperative to meticulously plan and implement a rigorous blinding protocol, such as employing a double-placebo method, while providing detailed descriptions of the methodology. Furthermore, all included studies were exclusively conducted in China. It is great need to enhance international collaboration in order to conduct multi-regional and multi-ethnic clinical studies that can validate the clinical efficacy of XSLJZD. Moreover, no follow-up assessments were conducted to determine the long-term effectiveness of XSLJZD. To precisely determine the clinical efficacy of XSLJZD for FD, further investigations with extended treatment and follow-up periods are warranted. The present review ultimately revealed a lack of description regarding quality control and chemical profile in all the studies included, thereby resulting in contentious conclusions. The future clinical trials on botanical drug extracts should provide comprehensive information regarding the composition, extraction process, and drug extraction ratio of the study material.

### 4.3 Comparison with previous studies

This review not only assessed the total clinical effectiveness rate, but also evaluated the enhancement in gastric emptying rate and symptom score, distinguishing it from previous studies (Zeng et al., 2013; He et al., 2018). The studies included in our analysis were limited to those that provided comprehensive details on the administration information and components of herbal prescriptions for the purpose of enhancing clarity. In addition, the scope of our search encompasses not only Chinese databases but also international databases, significantly expanding the time range for our investigation. Finally, we conducted subgroup and sensitivity analyses to assess result reliability and identify sources of heterogeneity. However, the limitation we face was the absence of Jadad rating in the literature compared with previous studies.

### 4.4 Advantages and future prospects

Compared to conventional Western medicine, XSLJZD demonstrated significant efficacy and a lower incidence of adverse events. Therefore, XSLJZD may serve as an effective complementary and alternative therapy for FD. Considering the suboptimal quality of the included studies, it is imperative to conduct robust large-scale RCTs in order to validate our findings. To enhance the evidential support for the efficacy of XSLJZD in patients with FD, well-designed RCTs should be undertaken, encompassing diverse outcome measures pertaining to dyspeptic symptoms. In addition, In order to enhance the robustness and reproducibility of findings, future studies on botanical drugs should provide detailed descriptions regarding their origin, composition, extraction process of metabolites, and drug ratios. Moreover, the efficacy of XSLJZD should be evaluated in the future studies with a long-term follow-up period.

## 5 Conclusion

By reviewing twenty-three RCTs, XSLJZD can be considered as effective and safe alternatives to FD. However, the validity of the included studies has been subject. Therefore, future research should focus on conducting more high-quality RCTs with standardized methodologies and rigorous designs.

## Data Availability

The original contributions presented in the study are included in the article, and all relevant data can be found in the article/Supplementary Material. The datasets for this study are uploaded to Figshare and it can be found on line at https://doi.org/10.6084/m9.figshare.24309844.
