# Supplementary material for: Efficacy and safety of Xiangsha liujunzi decoction for functional dyspepsia: a systematic review and meta-analysis
Source: Front Pharmacol. 2024 Jun 12;15:1356899. doi: 10.3389/fphar.2024.1356899 (PMC11200127; doi:10.3389/fphar.2024.1356899)
Supplement: Supplementary file 1 [file Table1.docx]

**Table S1. Characteristics of all eligible studies.**

| First author, year | Diagnostic criteria | Prescription (volume per intake, frequency; extraction process (n) | Species name, daily dosage | Control group (n) | Duration | Methodological characteristics | Outcome measures |
| --- | --- | --- | --- | --- | --- | --- | --- |
| Zhou, 2010 | Rome II | XSLJZD (200mL, b.i.d), decoction made from botanical drug mixtures (30) | Dried root of Codonopsis radix, 12g  Dried rhizome of *Atractylodis macrocephalae rhizoma*, 10g  Dried root of *Aucklandiae radix*, 6g  Dried ripe fruit of Amomi fructus, 6g  Dried pericarp of the ripe fruit of Citri reticulatae pericarpium, 10g  Dried sclerotium of Poria cocos, 10g  Dried tuber of Pinelliae rhizoma, 10g  Dried root and rhizome of Glycyrrhizae radix et rhizoma praeparata cum melle, 5g | Mosapride tablets 5mg t.i.d. (30) | 4weeks | Randomized controlled | A |
| Zhou et al., 2016 | Rome III | Modified XSLJZD (125mL, b.i.d), decoction made from botanical drug mixtures (60) | Dried root of Aucklandiae radix, 6g  Dried ripe fruit of Amomi fructus, 3g  Dried root of Codonopsis radix, 15g  Dried rhizome of Atractylodis macrocephalae rhizoma, 10g  Dried sclerotium of Poria cocos, 15g  Dried pericarp of the ripe fruit of Citri reticulatae pericarpium, 6g  Dried tuber of Pinelliae rhizoma, 10g  Dried root of Bupleuri radix, 10g  Dried root of Paeoniae radix alba, 10g  Dried tuber of Corydalis rhizoma, 10g  Dried stem bark, root bark or branch bark of Meliae cortex, 10g  Dried root and rhizome of Glycyrrhizae radix et rhizoma praeparata cum melle, 5g | Mosapride tablets 5mg t.i.d plus Lansoprazole enteric coated capsules 30mg q.d. (60) | 4 weeks | Randomized controlled | ACD |
| Yang et al., 2010 | Rome III | Modified XSLJZD (150mL, b.i.d), decoction made from botanical drug mixtures (30) | Dried root of Aucklandiae radix, 10g  Dried root of Codonopsis radix, 10g  Dried rhizome of Atractylodis macrocephalae rhizoma, 10g  Dried sclerotium of Poria cocos, 10g  Dried pericarp of the ripe fruit of Citri reticulatae pericarpium, 10g  Dried tuber of Pinelliae rhizoma, 10g  Dried immature fruit of Aurantii fructus, 10g  Dried root of Paeoniae radix alba, 10g  Dried root of Astragali radix, 30g  Dried ripe seed of Raphani semen, 15g  Dried root and rhizome of Glycyrrhizae radix et rhizoma praeparata cum melle, 10g | Domperidone tablets 10mg t.i.d. (30) | 4 weeks | Randomized controlled | AD |
| Xu et al., 2017 | Rome III | Modified XSLJZD (N.R, b.i.d), granules made from botanical drug mixtures (63) | Dried root of Aucklandiae radix, 10g  Dried ripe fruit of Amomi fructus, 6g  Dried root of Codonopsis radix, 15g  Dried rhizome of Atractylodis macrocephalae rhizoma, 15g  Dried sclerotium of Poria cocos, 15g  Dried pericarp of the ripe fruit of Citri reticulatae pericarpium, 12g  Dried tuber of Pinelliae rhizoma, 10g  Dried immature fruit of Aurantii fructus, 15g  Dried root and rhizome of Glycyrrhizae radix et rhizoma praeparata cum melle, 6g | Compound digestive enzyme capsule 2 tablets t.i.d. (62) | 4 weeks | Randomized controlled | ACD |
| Wang and Yang, 2016 | Rome III | XSLJZD (150mL, b.i.d), decoction made from botanical drug mixtures (60) | Dried root of Codonopsis radix, 18g  Dried rhizome of Atractylodis macrocephalae rhizoma, 15g  Dried root of Aucklandiae radix, 15g  Dried ripe fruit of Amomi fructus, 12g  Dried pericarp of the ripe fruit of Citri reticulatae pericarpium, 12g  Dried sclerotium of Poria cocos, 9g  Dried tuber of Pinelliae rhizoma, 6g  Dried root and rhizome of Glycyrrhizae radix et rhizoma praeparata cum melle, 6g | Domperidone tablets 10mg t.i.d. (60) | 8 weeks | Randomized controlled | AB |
| Wang et al., 2008 | Rome II | Modified XSLJZD (N.R, b.i.d), decoction made from botanical drug mixtures (30) | Dried root of Aucklandiae radix, 10g  Dried ripe fruit of Amomi fructus, 10g  Dried rhizome of Atractylodis macrocephalae rhizoma, 20g  Dried sclerotium of Poria cocos, 15g  Dried pericarp of the ripe fruit of Citri reticulatae pericarpium, 10g  Dried immature fruit of Aurantii fructus, 10g  Dried root of Paeoniae radix alba, 15g  Dried sandbag lining of Endothelium Corneum Gigeriae Galli, 15g  Dried germinated ripe fruit of Hordei fructus germinatus, 15g  Massa Medicata Fermentata, 10g  Dried tuber of Corydalis rhizoma, 15g  Dried fruit of Citri sarcodactylis fructus, 10g  Dried root and rhizome of Glycyrrhizae radix et rhizoma praeparata cum melle, 5g | Domperidone tablets 10mg t.i.d. (30) | 4 weeks | Randomized controlled | A |
| Wang, 2020 | Rome III | Modified XSLJZD (N.R, b.i.d), decoction made from botanical drug mixtures (50) | Dried root of Aucklandiae radix, 10g  Dried ripe fruit of Amomi fructus, 9g  Dried root of Ginseng radix et rhizoma, 9g  Dried rhizome of Atractylodis macrocephalae rhizoma, 15g  Dried sclerotium of Poria cocos, 15g  Dried pericarp of the ripe fruit of Citri reticulatae pericarpium, 10g  Dried tuber of Pinelliae rhizoma, 10g  Dried rhizome of Cyperi rhizoma, 10g  Dried tuber of Corydalis rhizoma, 10g  Dried ripe fruit of Jujubae fructus, 15g  Dried root of Paeoniae radix alba, 10g  Dried root and rhizome of Glycyrrhizae radix et rhizoma praeparata cum melle, 6g | Domperidone tablets 10mg t.i.d plus Esomeprazole enteric capsules 20mg q.d. (50) | 4 weeks | Randomized controlled | A |
| Sun, 2017 | Rome III | Modified XSLJZD (250mL, b.i.d), decoction made from botanical drug mixtures (50) | Dried root of Aucklandiae radix, 10g  Dried root of Codonopsis radix, 20g  Dried rhizome of Atractylodis macrocephalae rhizoma, 20g  Dried sclerotium of Poria cocos, 30g  Dried pericarp of the ripe fruit of Citri reticulatae pericarpium, 15g  Dried tuber of Pinelliae rhizoma, 15g  Dried rhizome of Dioscoreae rhizoma, 15g  Dried fruit of Crataegus monogyna Jacq., 10g  Massa Medicata Fermentata, 10g  Dried germinated ripe fruit of Hordei fructus germinatus, 10g  Dried root and rhizome of Glycyrrhizae radix et rhizoma praeparata cum melle, 5g | Domperidone tablets 10mg t.i.d. (50) | 4 weeks | Randomized controlled | AC |
| Liao, 2014 | Rome III | XSLJZD (150mL, b.i.d), decoction made from botanical drug mixtures (22) | Dried root of Aucklandiae radix, 10g  Dried ripe fruit of Amomi fructus, 10g  Dried root of Codonopsis radix, 20g  Dried rhizome of Atractylodis macrocephalae rhizoma, 15g  Dried sclerotium of Poria cocos, 15g  Dried pericarp of the ripe fruit of Citri reticulatae pericarpium, 10g  Dried tuber of Pinelliae rhizoma, 10g  Dried root and rhizome of Glycyrrhizae radix et rhizoma praeparata cum melle, 6g | Domperidone tablets 10mg t.i.d. (22) | 4 weeks | Randomized controlled | AC |
| Lu and Hong, 2012 | Rome III | Modified XSLJZD (N.R, b.i.d), decoction made from botanical drug mixtures (34) | Dried root of Aucklandiae radix, 15g  Dried ripe fruit of Amomi fructus, 10g  Dried root of Codonopsis radix, 15g  Dried rhizome of Atractylodis macrocephalae rhizoma, 12g  Dried sclerotium of Poria cocos, 20g  Dried pericarp of the ripe fruit of Citri reticulatae pericarpium, 10g  Dried tuber of Pinelliae rhizoma, 12g  Dried tuber of Corydalis rhizoma, 12g  Dried root and rhizome of Glycyrrhizae radix et rhizoma praeparata cum melle, 6g | Domperidone tablets 10mg t.i.d. (34) | 4 weeks | Randomized controlled | A |
| Liang, 2011 | Rome III | Modified XSLJZD (200mL, b.i.d), decoction made from botanical drug mixtures (30) | Dried root of Aucklandiae radix, 10g  Dried ripe fruit of Amomi fructus, 10g  Dried root of Codonopsis radix, 15g  Dried rhizome of Atractylodis macrocephalae rhizoma, 10g  Dried sclerotium of Poria cocos, 15g  Dried pericarp of the ripe fruit of Citri reticulatae pericarpium, 10g  Dried tuber of Pinelliae rhizoma, 10g  Dried stem bark, root bark or branch bark of Magnoliae officinalis cortex, 15g  Dried young fruit of Aurantii fructus immaturus, 15g  Dried root of Paeoniae radix alba, 10g  Dried root of Bupleuri radix, 10g  Dried root and rhizome of Glycyrrhizae radix et rhizoma praeparata cum melle, 10g | Mosapride tablets 5mg t.i.d. (32) | 4 weeks | Randomized controlled | AB |
| Li et al., 2022 | Rome IV | XSLJZD (N.R, t.i.d), granules made from botanical drug mixtures (26) | Dried root of Aucklandiae radix, 4g  Dried ripe fruit of Amomi fructus, 5g  Dried root of Codonopsis radix, 6g  Dried rhizome of Atractylodis macrocephalae rhizoma, 12g  Dried sclerotium of Poria cocos, 12g  Dried pericarp of the ripe fruit of Citri reticulatae pericarpium, 5g  Dried tuber of Pinelliae rhizoma, 6g  Dried root and rhizome of Glycyrrhizae radix et rhizoma praeparata cum melle, 4g | Placebo t.i.d. (28) | 4 weeks | Randomized controlled | ACD |
| Jiang, 2022 | Rome III | Modified XSLJZD (N.R, b.i.d), decoction made from botanical drug mixtures (80) | Dried root of Aucklandiae radix, 10g  Dried ripe fruit of Amomi fructus, 9g  Dried root of Ginseng radix et rhizoma, 10g  Dried rhizome of Atractylodis macrocephalae rhizoma, 15g  Dried sclerotium of Poria cocos, 18g  Dried pericarp of the ripe fruit of Citri reticulatae pericarpium, 12g  Dried tuber of Pinelliae rhizoma, 12g  Dried rhizome of Cyperi rhizoma, 15g  Dried tuber of Corydalis rhizoma, 12g  Dried root of Paeoniae radix alba, 10g  Dried ripe fruit of Jujubae fructus, 15g  Dried root and rhizome of Glycyrrhizae radix et rhizoma praeparata cum melle, 5g | Domperidone tablets 10mg t.i.d plus Esomeprazole enteric capsules 20mg q.d. (80) | 4 weeks | Randomized controlled | A |
| Cai et al., 2014 | Rome III | Modified XSLJZD (N.R, b.i.d), decoction made from botanical drug mixtures (27) | Dried root of Aucklandiae radix, 10g  Dried ripe fruit of Amomi fructus, 9g  Dried root of Ginseng radix et rhizoma, 10g  Dried rhizome of Atractylodis macrocephalae rhizoma, 15g  Dried sclerotium of Poria cocos, 18g  Dried pericarp of the ripe fruit of Citri reticulatae pericarpium, 12g  Dried tuber of Pinelliae rhizoma, 12g  Dried rhizome of Cyperi rhizoma, 15g  Dried tuber of Corydalis rhizoma, 12g  Dried root of Paeoniae radix alba, 10g  Dried ripe fruit of Jujubae fructus, 15g  Dried root and rhizome of Glycyrrhizae radix et rhizoma praeparata cum melle, 5g | Domperidone tablets 10mg t.i.d plus Esomeprazole enteric capsules 20mg q.d. (31) | 4 weeks | Randomized controlled | ACD |
| He, 2012 | Rome III | Modified XSLJZD (200mL, b.i.d), decoction made from botanical drug mixtures (30) | Dried root of Aucklandiae radix, 6g  Dried ripe fruit of Amomi fructus, 5g  Dried root of Codonopsis radix, 15g  Dried rhizome of Atractylodis macrocephalae rhizoma, 10g  Dried root of Astragali radix, 15g  Dried pericarp of the ripe fruit of Citri reticulatae pericarpium, 6g  Dried tuber of Pinelliae rhizoma, 10g  Dried root of Paeoniae radix alba, 10g  Dried immature fruit of Aurantii fructus, 6g  Dried root and rhizome of Glycyrrhizae radix et rhizoma praeparata cum melle, 5g | Mosapride tablets 5mg t.i.d plus Omeprazole Enteric Capsules 20mg q.d plus Pancreatin Capsules 300mg t.i.d. (30) | 6 weeks | Randomized controlled | A |
| Guo and Chen, 2017 | Rome III | Modified XSLJZD (200mL, b.i.d), decoction made from botanical drug mixtures (30) | Dried root of Aucklandiae radix, 10g  Dried ripe fruit of Amomi fructus, 10g  Dried root of Codonopsis radix, 20g  Dried rhizome of Atractylodis macrocephalae rhizoma, 15g  Dried sclerotium of Poria cocos, 15g  Dried pericarp of the ripe fruit of Citri reticulatae pericarpium, 10g  Dried tuber of Pinelliae rhizoma, 10g  Dried root of Paeoniae radix alba, 10g  Dried young fruit of Aurantii fructus immaturus, 15g  Dried root of Bupleuri radix, 12g  Dried rhizome of Cyperi rhizoma, 15g  Dried stem bark, root bark or branch bark of Magnoliae officinalis cortex, 15g  Dried root and rhizome of Salviae miltiorrhizae radix et rhizoma, 15g  Dried root and rhizome of Glycyrrhizae radix et rhizoma praeparata cum melle, 10g | Mosapride tablets 5mg t.i.d. (30) | 4 weeks | Randomized controlled | B |
| Feng, 2020 | Rome III | Modified XSLJZD (N.R, b.i.d), decoction made from botanical drug mixtures (75) | Dried root of Aucklandiae radix, 8g  Dried ripe fruit of Amomi fructus, 8g  Dried root of Codonopsis radix, 15g  Dried rhizome of Atractylodis macrocephalae rhizoma, 15g  Dried sclerotium of Poria cocos, 15g  Dried pericarp of the ripe fruit of Citri reticulatae pericarpium, 10g  Dried tuber of Pinelliae rhizoma, 10g  Dried root of Astragali radix, 30g  Dried root and rhizome of Glycyrrhizae radix et rhizoma praeparata cum melle, 6g | Jianwei Xiaoyan granule 20g t.i.d. (75) | 4 weeks | Randomized controlled | AD |
| Fan, 2010 | Rome III | Modified XSLJZD (250mL, b.i.d), decoction made from botanical drug mixtures (30) | Dried root of Aucklandiae radix, 6g  Dried ripe fruit of Amomi fructus, 3g  Dried root of Codonopsis radix, 10g  Dried rhizome of Atractylodis macrocephalae rhizoma, 12g  Dried sclerotium of Poria cocos, 15g  Dried pericarp of the ripe fruit of Citri reticulatae pericarpium, 6g  Dried tuber of Pinelliae rhizoma, 10g  Dried stem bark, root bark or branch bark of Magnoliae officinalis cortex, 10g  Dried immature fruit of Aurantii fructus, 10g  Dried root and rhizome of Glycyrrhizae radix et rhizoma praeparata cum melle, 6g | Domperidone tablets 10mg t.i.d. (30) | 4 weeks | Randomized controlled | AD |
| Cheng and Zhu, 2015 | Rome III | Modified XSLJZD (250mL, b.i.d), decoction made from botanical drug mixtures (40) | Dried root of Aucklandiae radix, 10g  Dried ripe fruit of Amomi fructus, 9g  Dried root of Ginseng radix et rhizoma, 10g  Dried rhizome of Atractylodis macrocephalae rhizoma, 15g  Dried sclerotium of Poria cocos, 20g  Dried pericarp of the ripe fruit of Citri reticulatae pericarpium, 12g  Dried tuber of Pinelliae rhizoma, 12g  Dried rhizome of Cyperi rhizoma, 15g  Dried ripe fruit of Jujubae fructus, 15g  Dried tuber of Corydalis rhizoma, 12g  Dried root of Paeoniae radix alba, 10g  Dried root and rhizome of Glycyrrhizae radix et rhizoma praeparata cum melle, 5g | Domperidone tablets 10mg t.i.d. (40) | 4 weeks | Randomized controlled | A |
| Chen, 2015 | Rome III | Modified XSLJZD (200mL, b.i.d), decoction made from botanical drug mixtures (75) | Dried root of Aucklandiae radix, 6g  Dried ripe fruit of Amomi fructus, 6g  Dried root of Codonopsis radix, 15g  Dried rhizome of Atractylodis macrocephalae rhizoma, 12g  Dried sclerotium of Poria cocos, 12g  Dried root of Astragali radix, 15g  Dried pericarp of the ripe fruit of Citri reticulatae pericarpium, 6g  Dried tuber of Pinelliae rhizoma, 6g  Dried young fruit of Aurantii fructus immaturus, 6g  Dried root tuber of Curcuma aromatica Salisb., 10g  Dried herb of Sarcandrae herba, 20g  Dried root and rhizome of Glycyrrhizae radix et rhizoma praeparata cum melle, 6g | Domperidone tablets 10mg t.i.d plus Vitamin B1 20mg t.i.d. (75) | 4 weeks | Randomized controlled | AD |
| Zeng and Tang, 2017 | Rome III | Modified XSLJZD (125mL, b.i.d), decoction made from botanical drug mixtures (44) | Dried root of Aucklandiae radix, 6g  Dried ripe fruit of Amomi fructus, 3g  Dried root of Codonopsis radix, 15g  Dried rhizome of Atractylodis macrocephalae rhizoma, 10g  Dried sclerotium of Poria cocos, 15g  Dried pericarp of the ripe fruit of Citri reticulatae pericarpium, 6g  Dried tuber of Pinelliae rhizoma, 10g  Dried root of Bupleuri radix, 10g  Dried root of Paeoniae radix alba, 10g  Dried tuber of Corydalis rhizoma, 10g  Dried stem bark, root bark or branch bark of Meliae cortex, 10g  Dried root and rhizome of Glycyrrhizae radix et rhizoma praeparata cum melle, 5g | Mosapride Tablets 5mg t.i.d plus Lansoprazole enteric coated capsules 30mg q.d. (44) | 4 weeks | Randomized controlled | A |
| Zhou et al., 2018 | Rome III | Modified XSLJZD (150mL, b.i.d), granules made from botanical drug mixtures (30) | Dried root of Aucklandiae radix, 6g  Dried ripe fruit of Amomi fructus, 3g  Dried root of Codonopsis radix, 10g  Dried rhizome of Atractylodis macrocephalae rhizoma, 10g  Dried sclerotium of Poria cocos, 10g  Dried pericarp of the ripe fruit of Citri reticulatae pericarpium, 6g  Dried tuber of Pinelliae rhizoma, 12g  Dried root of Bupleuri radix, 12g  Dried root of Paeoniae radix alba, 10g  Dried tuber of Corydalis rhizoma, 10g  Dried stem bark, root bark or branch bark of Meliae cortex, 10g  Dried root and rhizome of Glycyrrhizae radix et rhizoma praeparata cum melle, 3g | Mosapride Tablets 5mg t.i.d plus Rabeprazole enteric coated tablets 10mg q.d. (30) | 4 weeks | Randomized controlled | A |
| Lv et al., 2017 | Rome III | Modified XSLJZD (130mL, t.i.d), granules made from botanical drug mixtures (135) | Dried root of Astragali radix, N.R  Dried ripe fruit of Amomi fructus, N.R  Dried root of Codonopsis radix, N.R  Dried rhizome of Atractylodis macrocephalae rhizoma, N.R  Dried sclerotium of Poria cocos, N.R  Dried immature fruit of Aurantii fructus, N.R  Dried rhizome of Chuanxiong rhizoma, N.R  Dried tuber of Corydalis rhizoma, N.R  Massa Medicata Fermentata, N.R  Dried root and rhizome of Glycyrrhizae radix et rhizoma praeparata cum melle, N.R | Placebo t.i.d. (67) | 4 weeks | Randomized controlled | BCD |

Annotation: A= Clinical effective rate; B= Gastric emptying rate; C= Symptom score; D= Adverse reactions; N.R= not reported
